# Back Muscle Strength Is Associated with Self-Reported Morning-Erection Frequency in Apparently Healthy Japanese Male University Students: A Cross-Sectional Study

**DOI:** 10.3390/healthcare14060759

**Published:** 2026-03-18

**Authors:** Yoshiaki Endo, Takazo Tanaka, Kosuke Kojo, Chiaki Matsumoto, Masahiro Kurobe, Hiroyuki Nishiyama, Tatsuya Takayama, Jun Miyazaki

**Affiliations:** 1 Department of Physical Therapy, School of Health Science, International University of Health and Welfare, 2600-1 Kitakanemaru, Otawara 324-8501, Tochigi, Japan; yendo0117@ihwg.jp (Y.E.);; 2 Department of Urology, Tsukuba Gakuen Hospital, 2573-1 Kamiyokoba, Tsukuba 305-0854, Ibaraki, Japan; takazof43@gmail.com; 3 Department of Urology, Institute of Medicine, University of Tsukuba, 1-1-1 Tennodai, Tsukuba 305-8575, Ibaraki, Japan; 4 Tsukuba Clinical Research & Development Organization (T-CReDO), University of Tsukuba, 2-1-1 Amakubo, Tsukuba 305-8576, Ibaraki, Japan; 5 Center for Human Reproduction, International University of Health and Welfare Hospital, 537-3 Iguchi, Nasushiobara 329-2763, Tochigi, Japantakayama@ihwg.jp (T.T.); 6 Department of Systems Physiology, Graduate School of Medicine, University of the Ryukyus, 1076 Kiyuna, Ginowan 901-2720, Okinawa, Japan; 7 Department of Urology, School of Medicine, International University of Health and Welfare, 4-3 Kozunomori, Narita 286-8686, Chiba, Japan

**Keywords:** adolescent and young adult, body composition, erectile dysfunction, health behavior, lifestyle factors, muscle strength, nocturnal penile tumescence, reproductive health, sexual function, trunk muscles

## Abstract

**Highlights:**

**What are the main findings?**

- Back muscle strength, unlike handgrip strength or body mass index, is independently associated with increased morning-erection frequency in apparently healthy young men.
- Over 50% of healthy Japanese male university students report infrequent morning erections, suggesting that low nocturnal erectile indicators may be prevalent even in early adulthood.

**What are the implications of the main findings?**

- The frequency of morning erections serves as an intercourse-independent, low-burden indicator that could be useful for research and for initiating prevention-oriented conversations in university health services and primary care.
- Because this was a cross-sectional study and utilized a single-item self-reported outcome with modest discrimination, further longitudinal studies are needed before considering any screening or routine assessment applications.

**Abstract:**

**Background/Objectives**: Morning erections provide an intercourse-independent indicator of nocturnal erectile physiology. We aimed to examine whether body mass index (BMI) and muscle strength are associated with morning-erection frequency in apparently healthy Japanese male university students. **Methods**: This cross-sectional study analyzed 125 men with complete data (170 assessed; 45 excluded). Handgrip and back muscle strength were measured using dynamometry; BMI was calculated from height and weight. Morning-erection frequency was assessed using a single 6-category item and was dichotomized as low vs. high. Univariable and multivariable logistic regression models were fitted. Exploratory principal component analysis (PCA) and k-means clustering (*k* = 2, silhouette-supported) were performed. **Results**: Seventy-four participants (59.2%) were classified as low frequency. Back muscle strength was associated with high frequency (univariable odds ratio [OR] 1.61; 95% confidence interval [CI] 1.07–2.42; and *p* = 0.021) and remained significant after adjustment for BMI and handgrip strength (OR 1.88; 95% CI 1.02–3.47; and *p* = 0.045), whereas BMI and handgrip strength were not significant. Clustering identified two clusters (*n* = 41 and *n* = 84); Cluster 2 (higher BMI/strength) had a higher proportion of high morning-erection frequency (48% vs. 27%). **Conclusions**: In apparently healthy young men, greater back muscle strength was independently associated with higher self-reported morning-erection frequency. In this cohort, 59.2% reported infrequent morning erections, suggesting potential relevance even in early adulthood. Given the exploratory clustering, the single-item outcome, and likely residual confounding, these findings are hypothesis-generating and warrant longitudinal validation.

## 1. Introduction

Sexual and reproductive health constitutes a fundamental aspect of well-being throughout life and is increasingly recognized as relevant to public health and primary care. Erectile dysfunction (ED) is associated with cardiometabolic risk factors and adverse cardiovascular outcomes, suggesting that sexual health may serve as a potential marker of broader vascular and metabolic health in some contexts [1,2]. While ED is often discussed in middle-aged and older men, recent surveys conducted in Japan suggest that indices of sexual function may already be impaired in a substantial proportion of younger men [3,4]. Despite emerging evidence, reproductive and sexual health in young men remain relatively understudied in public health research and are often inadequately addressed in routine care.

Lifestyle and physical fitness are modifiable factors plausibly linked to sexual health. A systematic review and meta-analysis reported that physical activity and structured exercise are associated with reduced odds of ED [5]. Observational and interventional studies provide additional evidence for the associations between exercise/fitness and sexual function across adult populations [6,7,8]. However, much of the existing literature focuses on aerobic capacity or self-reported physical activity, whereas the role of muscle strength—particularly trunk-related strength—remains less understood in apparently healthy young men [9,10].

Sexual health assessment in younger men presents challenges due to the sensitivity of disclosure. Many erectile function questionnaires are centered on intercourse, complicating interpretation for sexually inexperienced or sexually inactive individuals and may be burdensome in non-specialist settings [11]. Recent research emphasizes that standard scales are inapplicable to men who have not engaged in vaginal intercourse, necessitating alternative, non-coital parameters for accurate assessment [12]. Furthermore, concentrating exclusively on intercourse-based metrics may disregard nuanced physiological changes or “neglected” aspects of sexual health, especially in demographics with low or solitary sexual activity [13]. Morning erections are commonly regarded as a pragmatic correlate of nocturnal penile tumescence and provide an intercourse-independent insight into nocturnal erectile physiology. In Japan, community-based studies have utilized nocturnal/morning-erection indicators to delineate patterns in sexual function [4]. Mixed-methods research in Japanese primary care revealed practical challenges in measuring male sexual dysfunction using self-report tools, underscoring the necessity for low-burden approaches [14]. Consequently, morning-erection frequency may provide an accessible proxy measure suitable for research in young men and as a starting point for prevention-oriented discussions in university health services and primary care.

In the present study, we aimed to examine whether two practical strength measures (handgrip strength and back muscle strength) and body mass index (BMI) are associated with morning-erection frequency in apparently healthy Japanese male university students. We also assessed the Erection Hardness Score (EHS) as a secondary metric to investigate the relationship between morning-erection frequency and erectile rigidity in this cohort. We hypothesized that greater trunk-related strength (back muscle strength) is associated with higher morning-erection frequency. We also performed exploratory principal component analysis (PCA) and clustering analyses to visualize integrated physical profiles.

## 2. Materials and Methods

### 2.1. Study Design and Setting

This exploratory cross-sectional observational study was conducted from July 2022 to July 2023 at the Otawara Campus of the International University of Health and Welfare (Otawara, Japan). The study aimed to generate preliminary insights into associations between muscle strength, BMI, and morning-erection frequency; therefore, a formal a priori sample size calculation was not performed.

### 2.2. Participants

Undergraduate students pursuing a major in physical therapy at the School of Health Sciences were recruited using convenience sampling. A total of 170 students were assessed for eligibility. Inclusion criteria were as follows: (i) male sex; (ii) age ≥18 years; and (iii) apparently healthy status. Exclusion criteria were as follows: (i) self-reported medical conditions that could affect male sexual function (e.g., hypertension, diabetes mellitus, and nephropathy); and (ii) incomplete questionnaire or measurement data.

Given the sensitive nature of sexual health questions, an explicit response option of “prefer not to answer” was included. Questionnaires that contained missing items or “prefer not to answer” responses for key variables were treated as missing. The primary analyses were conducted as a complete-case analysis, including participants with complete data for the variables required in the relevant models. To evaluate potential selection bias resulting from exclusions, we performed a supplementary comparison of baseline characteristics between included (complete cases) and excluded participants (those with medical conditions and/or missing or withheld responses).

### 2.3. Measurements

#### 2.3.1. Sexual Function Assessment

Morning-erection frequency was assessed using a single-item question: “How often do you notice a morning erection upon waking?” Responses were recorded on a six-point ordered scale: 1 (never), 2 (rarely), 3 (occasionally), 4 (every other day), 5 (frequently), and 6 (always). The wording and response structure were consistent with those employed in previous Japanese community-based research evaluating nocturnal/morning erections as a measure of male sexual function [4]. This item is conceptually related to the morning-erection frequency item included in the European Male Ageing Study Sexual Function Questionnaire (EMAS-SFQ; item 16), which assesses the frequency of waking with a full erection over the preceding month using a graded frequency scale [15]. Despite variations in response anchoring, both instruments aim to capture the perceived frequency of nocturnal/morning erections independently of sexual intercourse. Because formal psychometric validation data for this single-item measure in university-aged men are limited, we interpret morning-erection frequency as an exploratory proxy of nocturnal erectile function rather than a diagnostic measure.

For the primary analysis, morning-erection frequency was dichotomized a priori into low frequency (scores 1–3) and high frequency (scores 4–6), because a score of 4 (“every other day”) represents a face-valid threshold for regular morning erections. In complementary analyses, the original six-point scale was retained and analyzed as an ordinal outcome. Sensitivity analyses further assessed the robustness of the results against different dichotomization thresholds and the application of the full ordered outcome.

Erectile rigidity was additionally assessed using the EHS. The EHS grades erection hardness on a five-point scale: 0 (penis does not enlarge), 1 (penis is larger but not hard), 2 (penis is hard but not hard enough for penetration), 3 (penis is hard enough for penetration but not completely hard), and 4 (penis is completely hard and fully rigid). Following the classification applied in a recent Japanese national survey [3], participants were categorized into a “low/insufficient” group (grades 0–2), generally considered insufficient for penetration, and a “high/sufficient” group (grades 3–4). The EHS was included to examine convergent validity between perceived morning-erection frequency and erectile rigidity. Participants were not systematically screened for penetrative sexual experience; therefore, EHS responses may be hypothetical or speculative for some individuals who have little or no experience of such sexual intercourse.

To comprehensively characterize sexual function more broadly beyond mere erections, we collected three additional items from the sexual domain of the Aging Males’ Symptoms (AMS) questionnaire: perceived decline in sexual function (item 15), perceived decrease in morning erections (item 16), and perceived decline in sexual desire (item 17). Each item was rated on a five-point severity scale ranging from 1 (none) to 5 (extremely severe). Prior psychometric studies in Japanese populations have suggested that AMS items 12 and 14 may align with factors separate from the core sexual-symptom construct [16]; therefore, the present study focused on items 15–17, which more accurately represent subjective sexual decline. These items were analyzed individually as ordinal variables to capture complementary subjective dimensions of sexual function.

Furthermore, two items adapted from the International Index of Erectile Function (IIEF-15) were administered. Sexual desire frequency during the past month was assessed using an eight-category scale, with 1 indicating none and 8 representing two or more times per day. Although derived from the IIEF sexual desire domain (item 11), the response categories were modified to reflect absolute frequency rather than proportional occurrence. Conceptually, this item closely parallels the EMAS-SFQ item assessing frequency of sexual thoughts or interest (item 12). Satisfaction with sexual activity (including intercourse and/or masturbation) was assessed using a five-point scale from 1 (very dissatisfied) to 5 (very satisfied); this item is conceptually similar to the EMAS-SFQ item evaluating overall satisfaction with their sex life (item 19), although wording and scaling were adapted from the corresponding IIEF item (item 13) for the present study.

Finally, concern or distress regarding sexual function during the past month was assessed using a single-item question rated on a four-point scale from 1 (not at all) to 4 (very much). This item was adapted to encapsulate concern or distress related to sexual function broadly and conceptually corresponds to multiple EMAS-SFQ items addressing distress related to sexual activity (item 8), erectile ability (item 11), and morning erections (item 17). Owing to modifications from established instruments and having not been formally validated in university-aged men, all AMS-, IIEF-, and EMAS-derived measures were treated as exploratory.

All sexual-function questions included a “prefer not to answer” option; such responses were treated as missing, as previously described.

#### 2.3.2. Muscle Strength and Anthropometrics

Handgrip strength was measured using a digital dynamometer (Grip-D; TKK 5401; Takei Scientific Instruments Co., Ltd., Niigata, Japan). Two trials were performed for each hand, and the maximum value across both hands was utilized.

Back muscle strength was measured using a digital back dynamometer (Back-D; TKK 5402; Takei Scientific Instruments Co., Ltd., Niigata, Japan). Participants conducted two maximal trials; the highest value was used.

Height was assessed using a stadiometer and body mass using a digital scale. BMI was calculated as body mass (kg) divided by height squared (m^2^).

#### 2.3.3. Lifestyle Variables (Descriptive and Sensitivity Covariates)

Participants reported smoking status (never/past/current) and alcohol consumption frequency (days/week). Physical activity during the past 7 days was assessed using the International Physical Activity Questionnaire–Short Form (IPAQ-SF). The IPAQ-SF records the frequency (days/week) and duration (minutes/day) of vigorous-intensity activity, moderate-intensity activity, and walking, and sitting time during weekdays [17].

For IPAQ-SF processing, blank responses were assigned a value of 0 (indicating no activity). To mitigate the influence of extreme values, durations were truncated to a maximum of 180 min/day for each activity domain. Weekly physical activity was quantified as MET-min/week using standard MET values (vigorous = 8.0, moderate = 4.0, and walking = 3.3), calculated as (days/week) × (minutes/day) × (MET). Total physical activity was defined as the aggregate of vigorous, moderate, and walking MET-min/week. Participants were additionally categorized into IPAQ physical activity levels (low/moderate/high) using established categorical scoring criteria.

Dietary behaviors were evaluated using four single-item questions adapted from the National Health and Nutrition Survey in Japan [18] and validated chrononutrition instruments [19]: (i) weekly frequency of consuming three meals daily (0–7 days/week); (ii) weekly frequency of consuming meals combining a staple food, main dish, and side dish (SMS meals; 0–7 days/week), validated as a surrogate marker for nutrient adequacy in Japanese adults [20]; (iii) meal timing regularity (regular, sometimes irregular, or irregular); and (iv) frequency of eating or snacking after 21:00 (0–7 days/week), a threshold associated with adverse metabolic and cardiovascular outcomes [21].

Sleep habits were assessed using variables derived from the Munich Chronotype Questionnaire (MCTQ) [22]. Participants reported bedtime and wake-up time separately for weekdays and weekends. Sleep duration was calculated as the difference between wake-up time and bedtime, with bedtimes after midnight converted to a continuous time scale (e.g., 01:00 = 25:00) to accurately account for times spanning midnight. Social jetlag was calculated as the absolute difference between midpoint of sleep on weekends (MSF) and weekdays (MSW), SJL = |MSF − MSW|, where midpoint of sleep = bedtime + (sleep duration/2) [23].

These lifestyle variables and IPAQ-derived metrics were summarized descriptively. Smoking, alcohol consumption, and IPAQ-derived metrics served as covariates in sensitivity analyses; dietary and sleep timing variables were excluded as covariates to prevent potential overadjustment and model instability.

### 2.4. Statistical Analysis

Analyses focused on (a) correlations among anthropometric/strength parameters and morning-erection frequency, (b) primary binary logistic regression models utilizing standardized predictors, (c) sensitivity analyses to assess robustness of the primary association, and (d) exploratory PCA with k-means clustering to visualize integrated physical profiles.

Correlations: Spearman’s rank correlation coefficients were calculated among morning-erection frequency (six-point scale), EHS, BMI, handgrip strength, back muscle strength, height, body mass, and age. Given the strong conceptual and empirical relationship between BMI and body mass, BMI was used as the primary indicator of adiposity, and height/body mass were not concurrently included with BMI in the regression models.

Sensitivity analyses: Sensitivity analyses evaluated the relationship between back muscle strength and morning-erection frequency, including ordinal logistic regression for the original six-point outcome (proportional odds model) to assess robustness to dichotomization, as well as multivariable binary logistic regression additionally adjusted for age, smoking status, alcohol consumption frequency, and total physical activity (MET-min/week). Furthermore, as absolute strength correlates with overall body size, multivariable binary logistic regression using back muscle strength normalized to body mass was performed to assess whether the association reflects relative rather than absolute strength.

In additional outcome-definition sensitivity analyses, the dichotomization threshold for morning-erection frequency was varied sequentially (score 6 vs. 1–5, scores 5–6 vs. 1–4, scores 4–6 vs. 1–3 [primary], scores 3–6 vs. 1–2, and scores 2–6 vs. 1). For each threshold, we fitted (i) a back-only binary logistic regression model and (ii) a multivariable model including BMI, handgrip strength, and back muscle strength (all z-score standardized), summarizing discriminative ability using the area under the receiver operating characteristic curve (AUC). AUC 95% confidence intervals were computed using DeLong’s method.

Exploratory profiling: PCA was conducted on standardized BMI, handgrip strength, and back muscle strength values to facilitate visualization, and participants were partitioned using k-means clustering. The optimal number of clusters (*k*) was determined based on silhouette analysis across *k* = 2–6.

For exploratory comparison, a composite physical profile score was established as the first principal component (PC1) obtained from PCA of z-score-standardized BMI, handgrip strength, and back muscle strength; the PC1 direction was oriented so that higher values correspond to higher back muscle strength. The association between PC1 and the primary binary outcome was evaluated using logistic regression, and the discrimination (AUC) was compared with the back-only model. AUCs were calculated from fitted probabilities (apparent discrimination).

All analyses were conducted using R (version 4.3.2; R Foundation for Statistical Computing, Vienna, Austria). Two-tailed tests were used, and the significance level was set at *p* < 0.05. Given the exploratory nature and multiple sensitivity/outcome-definition analyses, *p*-values should be considered nominal and were not adjusted for multiplicity. Group comparisons for continuous variables and ordinal outcomes, including morning-erection frequency, analyzed on the original six-point scale and EHS, were conducted using the Brunner–Munzel test. Categorical variables, including dichotomized outcomes, were analyzed using Barnard’s exact test for 2 × 2 contingency tables and Fisher’s exact test for 2 × 3 contingency tables.

### 2.5. Ethics

The study protocol was approved by the Institutional Review Board of the International University of Health and Welfare (approval number: 21-Io-42; date of approval: 28 May 2022). The study was conducted in accordance with the Declaration of Helsinki. Participants provided informed consent via an online form before commencing the survey, and again upon completion, after being informed of the study’s purpose and data management procedures.

## 3. Results

### 3.1. Participant Characteristics

A total of 170 men were enrolled. After excluding 45 participants because of missing required measurements (BMI and/or muscle strength), self-reported medical history potentially affecting sexual function, and/or missing or withheld responses (including “prefer not to answer”) to key questionnaire items, 125 with complete data were included in the analysis; the participant flow is illustrated in Figure S1. Baseline characteristics of included and excluded participants are summarized in Tables S1 and S2. Included participants were marginally older than those excluded (20.09 ± 1.17 vs. 19.62 ± 0.72 years; *p* = 0.013), although morning-erection frequency, EHS, and other sexual variables were largely comparable between groups.

Participant characteristics stratified by the morning-erection frequency group are shown in Table 1, Tables S3 and S4. Table 2 presents the distribution of morning-erection frequency scores. Overall, 74 participants (59.2%) were classified as low frequency (scores 1–3), and 51 (40.8%) as high frequency (scores 4–6). Regarding erectile rigidity, the majority of participants maintained sufficient function. According to the EHS classification, 118 participants (94.4%) were categorized in the high/sufficient group (Grades 3–4), while only 7 (5.6%) were categorized into the low/insufficient group (Grades 0–2) (Table 2). The distribution of the additional sexual variables is shown in Table S5; most participants reported no or mild perceived sexual decline. Notably, sexual desire frequency was heterogeneous, with 37.6% (47/125) indicating no sexual desire in the past month, whereas concern or distress regarding sexual function was predominantly low (75.2%; 94/125, “not at all”).

Back muscle strength (kg) was greater in the high-frequency group than in the low-frequency group (108.53 ± 28.49 vs. 119.78 ± 21.00; *p* = 0.015), whereas BMI and handgrip strength did not differ significantly (Table 1). Sleep variables were summarized descriptively; in these exploratory comparisons, weekday bedtime was slightly later in the high-frequency group (01:03 ± 65 vs. 00:24 ± 96; *p* = 0.027), although other sleep timing and duration metrics showed no significant differences (Table S4).

### 3.2. Correlation Analyses

Morning-erection frequency exhibited a positive correlation with back muscle strength (rs = 0.256, *p* = 0.004). Furthermore, a weak but significant positive correlation was observed between morning-erection frequency and EHS (rs = 0.201, *p* = 0.025). However, EHS was not significantly correlated with back muscle strength (rs = −0.150, *p* = 0.096) or handgrip strength (rs = −0.082, *p* = 0.366). Correlations with BMI, handgrip strength, height, body mass, and age were not significant. The full correlation matrix is provided in Table S6.

In exploratory correlations, morning-erection frequency was not significantly correlated with sexual desire frequency or satisfaction with sexual activity (Table S7). Similarly, back muscle strength exhibited no significant correlations with subjective sexual function variables, including the perceived decline in sexual function or sexual distress (Table S8). These findings suggest that the association between back muscle strength and morning erections likely represents a physiological connection rather than psychological or libido-driven factors.

### 3.3. Logistic Regression Analyses

**Figure 1:**
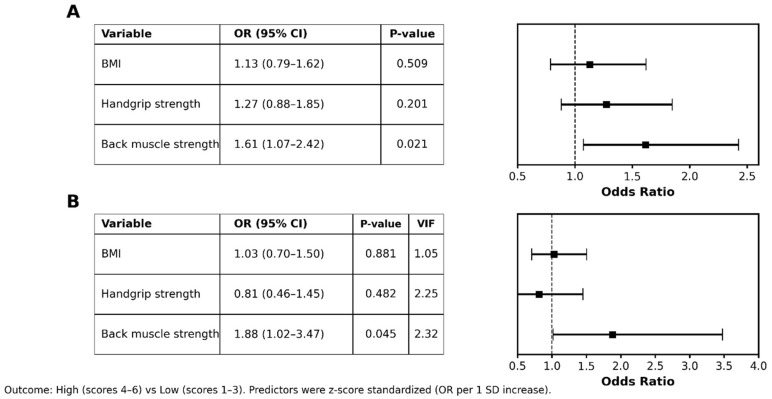
Logistic regression analyses of factors associated with morning-erection frequency. (**A**) Univariable logistic regression for BMI, handgrip strength, and back muscle strength. (**B**) Multivariable logistic regression including BMI, handgrip strength, and back muscle strength. Morning-erection frequency is dichotomized into low (scores 1–3) and high (scores 4–6) groups. Predictors are z-score standardized; ORs represent changes per 1 SD increase. BMI, body mass index; VIF, variance inflation factor; CI, confidence interval; OR, odds ratio; and SD, standard deviation.

In univariable logistic regression (OR per 1 SD increase), back muscle strength was positively associated with high morning-erection frequency (OR 1.61; 95% CI 1.07–2.42; and *p* = 0.021), whereas BMI and handgrip strength did not show a significant relationship (Figure 1A).

In the multivariable model including BMI, handgrip strength, and back muscle strength, only back muscle strength showed a significant association (OR 1.88; 95% CI 1.02–3.47; and *p* = 0.045) with acceptable multicollinearity (VIFs ≤ 2.32) (Figure 1B). Discrimination was modest for both the back-only model (AUC 0.625; 95% CI 0.525–0.724) and the multivariable model (AUC 0.633; 95% CI 0.534–0.732) regarding the primary dichotomization (scores 4–6 vs. 1–3). Results of discrimination under alternative outcome dichotomizations are summarized in Table S9.

To assess potential confounding and to inform covariate selection for sensitivity analyses, associations of age, smoking status, alcohol consumption frequency, and physical activity metrics derived from the IPAQ-SF with the primary binary outcome are presented in Table S10. Among these, walking activity (per 1000 MET-min/week) showed a modest positive association with high morning-erection frequency (OR 1.25; 95% CI 1.01–1.55; and *p* = 0.037). In contrast, age, smoking status, alcohol consumption frequency, vigorous exercise frequency (sessions/week), vigorous and moderate activity (MET-min/week), total physical activity (MET-min/week), sitting time, and IPAQ activity level were not significantly associated with the outcome.

Given that these variables are biologically plausible confounders, they were additionally adjusted for in sensitivity analyses. In particular, the lifestyle-adjusted model incorporated total physical activity (MET-min/week) derived from the IPAQ-SF to reflect overall physical activity exposure.

### 3.4. Sensitivity Analyses

Sensitivity analyses aligned directionally with the primary findings. Ordinal logistic regression for the original six-category outcome indicated that back muscle strength remained positively associated with morning-erection frequency (OR 1.88; 95% CI 1.14–3.09; and *p* = 0.013). After additional adjustment for age and lifestyle variables (smoking status and alcohol consumption frequency), with physical activity quantified as total physical activity derived from the IPAQ-SF (per 1000 MET-min/week), the association between back muscle strength and high morning-erection frequency remained significant (OR 1.87; 95% CI 1.00–3.49; and *p* = 0.049). Analyses utilizing back muscle strength normalized to body mass also showed a consistent association (OR 2.09; 95% CI 1.11–3.93; and *p* = 0.022). These results are summarized in Figure S3.

The results remained broadly consistent across various dichotomization thresholds of morning-erection frequency, demonstrating modest discrimination (Table S9).

### 3.5. Exploratory PCA and Clustering

**Figure 2:**
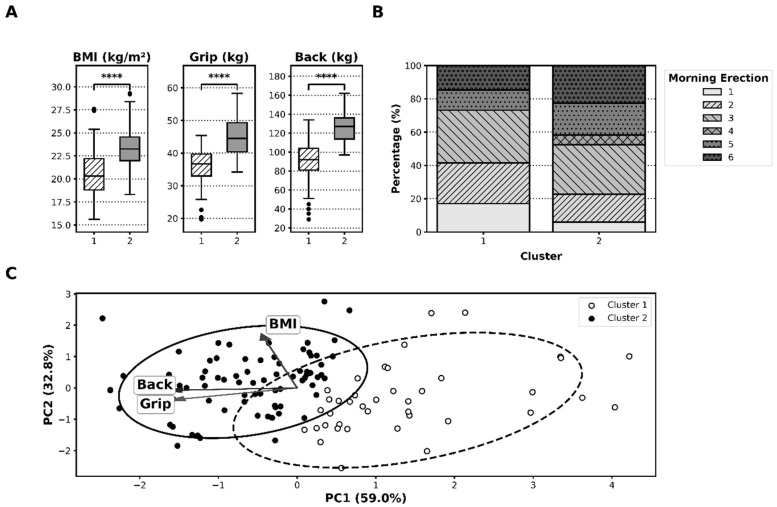
Results of exploratory analysis (PCA + k-means). The groups represent data-driven k-means clusters based on standardized BMI, handgrip, and back strength; *k* was chosen by silhouette analysis. (**A**) Differences in BMI, handgrip strength, and back muscle strength between Clusters 1 and 2. ****, *p* < 0.001. (**B**) Distribution of morning-erection frequency (six-point scale) across clusters. (**C**) PCA scatter plot showing partial separation of clusters, with arrows indicating contributions of BMI and strength variables. BMI, body mass index; PCA, principal component analysis.

Silhouette analysis across *k* = 2–6 supported the selection of *k* = 2 clusters (Figure S2). K-means clustering (*k* = 2) applied to standardized BMI, handgrip strength, and back muscle strength resulted in the identification of two clusters: Cluster 1 (*n* = 41) and Cluster 2 (*n* = 84). Cluster 2 exhibited a significantly higher BMI, handgrip strength, and back muscle strength than Cluster 1 (all *p* < 0.001; Table 3; Figure 2A).

The proportion of participants exhibiting high morning-erection frequency was higher in Cluster 2 than in Cluster 1 (48% vs. 27%; *p* = 0.026) (Figure 2B). PCA visualization showed the partial separation of clusters; PC1 primarily reflected strength, while PC2 primarily reflected BMI (Figure 2C).

Additional participant characteristics by cluster, including age, height, body mass, smoking status, alcohol consumption frequency, and IPAQ-derived physical activity metrics (vigorous/moderate/walking/total MET-min/week, sitting time on weekdays, and IPAQ physical activity level), are provided in Table S11. As shown in the table, moderate-intensity physical activity (MET-min/week) was higher in Cluster 1 than in Cluster 2 (*p* = 0.015). However, no significant differences were observed between the clusters for vigorous activity days, vigorous activity (MET-min/week), total physical activity (MET-min/week), and IPAQ physical activity level. In an exploratory logistic regression, the PCA-derived composite physical profile score (PC1, oriented so that higher values correspond to higher back muscle strength) was associated with increased morning-erection frequency; however, discrimination was comparable to the back-only model (Table S12).

Sensitivity analyses corroborated the primary findings, including ordinal logistic regression using the full six-category outcome, additional adjustment for lifestyle covariates, and analyses utilizing body mass-normalized back strength (Figure S3). The results were also broadly consistent across alternative dichotomization thresholds, with modest discrimination (Table S9).

## 4. Discussion

### 4.1. Principal Findings

In this cross-sectional study of apparently healthy Japanese male university students, back muscle strength was independently and positively associated with self-reported morning-erection frequency. Notably, 59.2% of participants reported infrequent morning erections (scores 1–3), suggesting that low morning-erection frequency may be prevalent even among young men who appear to be healthy.

### 4.2. Interpretation in the Context of Prior Work

Sexual health is increasingly recognized as interconnected with cardiometabolic health and overall well-being [1,2]. Although ED is often framed as a condition for older age, survey data from Japan suggest that indices of sexual function may already be impaired in younger men [3,4]. However, sexual health assessment in this demographic is complicated. Gold-standard instruments like the IIEF are often inappropriate for sexually inexperienced men, a demographic constituting nearly half of young men seeking assessment [24]. While masturbation-based metrics provide better sensitivity in this population [12], morning-erection frequency functions as a valuable, intercourse-independent indicator for public health and primary care research [14]. In our study, morning-erection frequency significantly correlated with EHS, supporting its convergent validity as a proxy for erectile function, despite the potential for hypothetical EHS ratings among inexperienced participants.

Previous studies have emphasized the importance of aerobic fitness and physical activity [5,6,7,8]. Evidence regarding muscle strength—particularly trunk-related strength—in healthy young men is limited [9]. Studies involving older or clinical populations have reported associations between handgrip strength, gait/physical function, and ED severity [25,26,27], consistent with broader links between physical function and erectile physiology [28]. In contrast, in our young and apparently healthy cohort, back muscle strength showed the most pronounced association, whereas handgrip strength was not significantly related to morning-erection frequency.

Measures of strength have also been linked to cardiometabolic risk profiles in population studies [29,30], supporting the notion that trunk-related strength may correlate with broader physiological status relevant to nocturnal erectile function. However, we did not measure cardiometabolic biomarkers, sleep architecture, or hormones; thus, mechanistic interpretation remains speculative.

Emerging evidence in adult men suggests that reduced skeletal muscle mass [31] and poorer muscle quality [10] may be associated with erectile dysfunction and treatment responsiveness. Although our study did not measure muscle mass/quality or hormones, our findings are directionally consistent with this evolving evidence and support further mechanistic and longitudinal research. Additionally, current international guidelines (such as those from the European Association of Urology [32] and the American Urological Association [33]) advocate for lifestyle modifications and physical fitness as foundational management strategies for ED, further underscoring the clinical relevance of investigating physical strength as a correlate of sexual health.

### 4.3. Potential Explanatory Pathways and Alternative Explanations

Morning erections reflect nocturnal penile tumescence and are contingent upon neurovascular and hormonal physiology. Trunk strength activities require the coordinated neuromuscular function of large muscle groups and may serve as markers of overall conditioning, recuperation, and health behaviors rather than direct causal determinants of erectile physiology. The training of the diaphragm and abdominal musculature has been reported to influence pelvic floor strength and endurance [34], suggesting a potential neuromuscular linkage between trunk-related function and erectile physiology.

While highly speculative given the modest effect size observed, two additional physiological pathways could theoretically contribute to this association. First, skeletal muscles secrete myokines that promote angiogenesis and nitric oxide (NO) production [35,36]. Since nocturnal erections are NO-dependent [37], large muscle mass might conceivably support penile endothelial integrity. Second, trunk posture and paraspinal muscle tone can influence autonomic balance [38]. Since nocturnal erections require parasympathetic dominance [39], adequate back muscle strength might hypothetically reduce paraspinal tension, potentially assisting the autonomic transition required for nocturnal tumescence.

Despite the lack of hormonal data, the role of testosterone warrants consideration. Nocturnal penile tumescence (NPT) is androgen-dependent but exhibits a “threshold effect,” wherein the frequency and magnitude do not always correspond linearly with testosterone levels once they surpass the hypogonadal range [40,41]. In apparently healthy students, testosterone levels likely fall within a range where hormonal variation exerts a limited direct influence on NPT frequency. Moreover, muscle strength in young adults often depends more on neural parameters (e.g., motor unit recruitment) than solely on hormonal concentrations [42,43].

The specific association of morning erections with back strength as opposed to handgrip strength or BMI reinforces this perspective. While handgrip strength correlates with testosterone in some populations [44,45], this link is inconsistent in healthy students [46,47]. Our findings suggest that trunk-specific neuromuscular or biomechanical factors may play a distinct role beyond general hormonal influence.

Moreover, psychological stress can influence nocturnal erections, and psychogenic pathways involving autonomic and hypothalamic–pituitary–adrenal axis activity are being implicated in psychogenic ED [48]. These factors were not measured in the present study and may have contributed to the observed association. BMI also does not differentiate between fat mass and lean mass; in this cohort, the higher BMI observed in the stronger cluster may partly reflect greater lean mass rather than adiposity; however, since body composition was not assessed, future studies should consider incorporating direct assessment of body composition and fat distribution.

We also emphasize that the observed effect is insufficient for individual-level classification. Thus, even if back strength is associated with morning-erection frequency, it is not suitable as an independent screening parameter for individuals in this dataset.

### 4.4. Characteristics of the Study Population and Generalizability

Our participants were physical therapy students, who typically exhibit higher levels of physical activity and health literacy. Previous international studies report that physical therapy students are significantly more active than those from other disciplines [49,50,51,52]. Similarly, Japanese rehabilitation students tend to have high health consciousness [53].

However, our cohort’s physical activity (mean ~2300 MET-min/week) was considerably lower than these international benchmarks and more comparable to student populations with reduced activity levels [54]. The participants exhibit a diverse range of activity levels (low: 35.2%; moderate: 35.2%; and high: 29.6%), indicating that our sample is not exclusively composed of a high-fitness population, thus maintaining some comparability to the general young male population.

Critically, despite the potential for a “healthy student effect” inherent in this major, nearly 60% of our participants reported low morning-erection frequency. If early indications of nocturnal erectile decline are prevalent even in this relatively health-conscious and educated group, the prevalence in the general population—potentially characterized by lower health awareness and physical activity—may even be higher. Thus, the association between back muscle strength and morning erections observed in this context may have broader implications for preventative health strategies in young men.

### 4.5. Public Health and Primary Care Implications

From a public health and primary care perspective, two points are relevant. First, the significant prevalence of low morning-erection frequency among ostensibly healthy young men suggests that nocturnal erectile indicators may manifest earlier than commonly assumed [3,4]. Second, morning-erection frequency serves as an intercourse-independent and low-burden indicator, potentially offering a more feasible alternative to conventional intercourse-centered questionnaires for some young men. This measure may facilitate the initiation of prevention-oriented discussions regarding sleep, stress, and lifestyle behaviors in university health services, occupational health settings, and primary care [14].

However, the multivariable model yielded an AUC of 0.63, indicating poor discriminative ability. Therefore, these findings do not support using back muscle strength as a routine screening or diagnostic tool for individuals. Instead, they should be considered hypothesis-generating and relevant for guiding future longitudinal and interventional research.

Because sexual health data are sensitive, any use of morning-erection inquiries in health services or surveys must be voluntary, privacy-preserving, and explicitly presented as prevention-oriented and non-diagnostic. Moreover, because morning-erection frequency was assessed using a single self-reported item without age-specific psychometric validation, prevalence estimates and effect sizes should be interpreted as exploratory.

### 4.6. Strengths and Limitations

The strengths of this study include the focus on an understudied population (young men), the use of an intercourse-independent outcome, objective dynamometry, and robust sensitivity analyses.

The following limitations should also be noted:

1. Causal Inference: The cross-sectional design precludes causal conclusions.
2. Sampling: Convenience sampling from a single university limits generalizability. No a priori sample size calculation was performed; some estimates were imprecise, several *p*-values were close to the conventional threshold, and confidence intervals were wide, suggesting limited power for modest effects. Therefore, findings should be interpreted with caution and require replication.
3. Measurement: Morning-erection frequency was assessed via a single self-reported item without specific psychometric validation for this age group, leaving room for potential recall or social desirability biases. Validation studies using established multi-item questionnaires or objective NPT measures (e.g., RigiScan^®^) in a subset are warranted.
4. Data Completeness: Substantial exclusions (26.5%) due to missing data or medical history may introduce selection bias. Additional comparisons suggested that excluded participants were slightly younger, though further analyses were constrained by missing data.
5. EHS Ceiling Effect: A ceiling effect was observed in EHSs (94.4% sufficient hardness). As EHS anchors refer to penetration, sexually inexperienced participants likely provided hypothetical ratings, introducing measurement error and limiting the evaluation of strength–rigidity associations.
6. Unmeasured Confounders: We did not measure sleep quality, psychological stress (including academic stress), diet, medication/supplements use, objective aerobic fitness, or cardiometabolic/hormonal biomarkers. Reports have suggested a decline in serum testosterone levels among adolescent and young adult men in some settings [55,56], but we did not assess hormones.
7. Sensitive Nature of Data: Despite the “prefer not to answer” option, nonresponse to sexual health questions may have been non-random, the bias of which is essentially indeterminate.
8. Socioeconomic and Relationship Factors: Socioeconomic and relationship factors, known to influence sexual function [57], were not assessed.

### 4.7. Future Directions

Future longitudinal studies should evaluate whether changes in trunk-related strength, physical activity, sleep, stress, hormonal/cardiometabolic profiles, and psychosocial factors can predict changes in morning-erection frequency over time. Interventional studies may help evaluate the impact of lifestyle interventions (exercise training, sleep improvement, and stress reduction) on morning-erection frequency and associated health outcomes. Broader sampling across diverse socioeconomic and cultural contexts is needed for developing generalizable public health and primary care strategies.

## 5. Conclusions

In apparently healthy Japanese male university students, back muscle strength is independently associated with morning-erection frequency, whereas BMI and handgrip strength are not. More than half of the participants report infrequent morning erections, suggesting that reduced nocturnal erectile indicators may be relatively prevalent even among young men. Given the single-item self-reported outcome, substantial exclusions, and likely residual confounding, these findings should be interpreted as hypothesis-generating rather than diagnostic. Further longitudinal and interventional research is warranted to inform prevention-oriented approaches in public health and primary care.

## Figures and Tables

**Table 1:** Body mass index, muscle strength, and lifestyle characteristics stratified by morning-erection frequency group (*n* = 125).

Variable	Total (*n* = 125)	Low (1–3) (*n* = 74)	High (4–6) (*n* = 51)	*p*-Value
Age (years)	20.09 ± 1.17	20.07 ± 1.33	20.12 ± 0.91	0.206
Body mass index (kg/m^2^)	22.33 ± 2.66	22.20 ± 2.81	22.52 ± 2.44	0.542
Handgrip strength (kg)	41.77 ± 7.61	41.05 ± 7.70	42.83 ± 7.41	0.302
Back muscle strength (kg)	113.12 ± 26.20	108.53 ± 28.49	119.78 ± 21.00	**0.01** **5**
Alcohol consumption frequency (days/week)	0.48 ± 0.87	0.45 ± 0.91	0.53 ± 0.81	0.344
Smoking status, *n* (%)				0.421
Never smoked	101 (80.8%)	60 (81.1%)	41 (80.4%)	
Past smoker	4 (3.2%)	1 (1.4%)	3 (5.9%)	
Current smoker	20 (16.0%)	13 (17.6%)	7 (13.7%)	
Total physical activity (MET-min/week)	2308.5 ± 2168.4	2285.4 ± 2229.0	2342.0 ± 2098.7	0.769

Values are presented as mean ± standard deviation (SD) or *n* (%). *p*-values were obtained using the Brunner–Munzel test for continuous variables. For categorical variables, Fisher’s exact test was used. Body mass index (BMI) was calculated as body mass (kg)/height (m^2^). Bold values indicate statistical significance (*p* < 0.05).

**Table 2:** Distribution of sexual function parameters among participants (*n* = 125).

Variable/Score		*n*	%
Morning-erection frequency			
	1 (Never)	12	9.6
	2 (Rarely)	24	19.2
	3 (Occasionally)	38	30.4
	4 (Every other day)	5	4.0
	5 (Frequently)	21	16.8
	6 (Always)	25	20.0
EHS			
	0 (Not enlarged)	1	0.8
	1 (Larger but not hard)	2	1.6
	2 (Hard but not enough for penetration)	4	3.2
	3 (Hard enough for penetration)	16	12.8
	4 (Completely hard and fully rigid)	102	81.6

The mean ± SD for morning-erection frequency (1–6) and EHS (0–4) are 3.59 ± 1.66 and 3.73 ± 0.68, respectively. EHS, Erection Hardness Score; SD, standard deviation.

**Table 3:** Comparison of characteristics between clusters.

Characteristic	Cluster 1 (*n* = 41)	Cluster 2 (*n* = 84)	*p*-Value
BMI (kg/m^2^)	20.62 ± 2.64	23.17 ± 2.25	**<0.001**
Handgrip strength (kg)	35 ± 7	45 ± 6	**<0.001**
Back muscle strength (kg)	87 ± 25	126 ± 16	**<0.001**
Morning-erection frequency	3.10 ± 1.67	3.82 ± 1.61	**0.018**
Morning-erection frequency, *n* (%)			**0.0** **27**
Low (≤3)	30 (73%)	44 (52%)	
High (≥4)	11 (27%)	40 (48%)	

Values are presented as mean ± standard deviation or *n* (%). Body mass index (BMI) is calculated as body mass (kg)/height (m^2^). *p*-values are obtained using the Brunner–Munzel test for continuous variables and the Barnard exact test for categorical variables. Bold values indicate statistical significance (*p* < 0.05).

## Data Availability

The data presented in this study are available from the corresponding author upon reasonable request due to privacy and ethical restrictions.

## Supplementary Materials

The following supporting information can be downloaded at: https://www.mdpi.com/article/10.3390/healthcare14060759/s1. Additional File S1 (Supplementary Materials), including Figure S1: Flow diagram of participant enrollment; Figure S2: Silhouette analysis for k-means clustering; Figure S3: Sensitivity analyses for the association between back muscle strength and morning-erection frequency; Table S1: Comparison of baseline characteristics between included and excluded participants; Table S2: Comparison of sleep habits between included and excluded participants; Table S3: Additional participant characteristics and sexual function variables stratified by morning-erection frequency group (*n* = 125); Table S4: Sleep habits stratified by morning-erection frequency group among included participants (*n* = 125); Table S5: Distribution of additional sexual function variables among participants (*n* = 125); Table S6: Spearman’s correlation coefficients among anthropometric strength and morning-erection frequency variables (*n* = 125); Table S7: Spearman’s correlation coefficients among sexual function variables (*n* = 125); Table S8: Spearman’s correlation coefficients among anthropometric strength and additional sexual variables (*n* = 125); Table S9: Sensitivity analyses across alternative dichotomizations of morning-erection frequency; Table S10: Univariable associations of age and lifestyle variables with high morning erection frequency (scores 4–6 vs. 1–3); Table S11: Additional participant characteristics by k-means cluster; Table S12: Exploratory logistic regression using PCA-derived composite physical profile score (PC1) and discrimination comparison.

## Abbreviations

The following abbreviations are used in this manuscript:

AMS	Aging Males’ Symptoms
AUC	Area under the receiver operating characteristic curve
BMI	Body mass index
CI	Confidence interval
ED	Erectile dysfunction
EHS	Erection Hardness Score
EMAS-SFQ	European Male Ageing Study Sexual Function Questionnaire
IIEF	International Index of Erectile Function
IPAQ-SF	International Physical Activity Questionnaire–Short Form
NO	Nitric oxide
NPT	Nocturnal penile tumescence
MCTQ	Munich Chronotype Questionnaire
MET	Metabolic equivalent of task
OR	Odds ratio
PC1	First principal component
PCA	Principal component analysis
SD	Standard deviation
VIF	Variance inflation factor
