# Supplementary material for: Back Muscle Strength Is Associated with Self-Reported Morning-Erection Frequency in Apparently Healthy Japanese Male University Students: A Cross-Sectional Study
Source: Healthcare (Basel). 2026 Mar 18;14(6):759. doi: 10.3390/healthcare14060759 (PMC13027116; doi:10.3390/healthcare14060759)
Supplement: Supplementary file 1 [file healthcare-14-00759-s001.zip › Additional_File_S1_20260128_1442.pdf]

Additional File

Back Muscle Strength Is Associated with Self-Reported Morning-Erection Frequency in Apparently Healthy Japanese Male University Students: A Cross-Sectional Study

Yoshiaki Endo, Takazo Tanaka, Kosuke Kojo, Chiaki Matsumoto, Masahiro Kurobe, Hiroyuki Nishiyama, Tatsuya Takayama, and Jun Miyazaki

Additional File S1: Supplementary materials

Page 2.	Figure S1.	Flow diagram of participant enrollment.
Page 3.	Figure S2.	Silhouette analysis for k-means clustering.
Page 4.	Figure S3.	Sensitivity analyses for the association between back muscle strength and morning-erection frequency.
Page 5.	Table S1.	Comparison of baseline characteristics between included and excluded participants.
Page 6.	Table S2.	Comparison of sleep habits between included and excluded participants.
Page 7.	Table S3.	Additional participant characteristics and sexual function variables stratified by morning-erection frequency group (n = 125).
Page 8.	Table S4.	Sleep habits stratified by morning-erection frequency group among included participants (n = 125).
Page 9.	Table S5.	Distribution of additional sexual function variables among participants (n = 125).
Page 10.	Table S6.	Spearman's correlation coefficients among anthropometric strength and morning-erection frequency variables (n = 125).
Page 11.	Table S7.	Spearman's correlation coefficients among sexual function variables (n = 125).
Page 12.	Table S8.	Spearman's correlation coefficients among anthropometric strength and additional sexual variables (n = 125).
Page 13.	Table S9.	Sensitivity analyses across alternative dichotomizations of morning-erection frequency.
Page 14.	Table S10.	Univariable associations of age and lifestyle variables with high morning erection frequency (scores 4–6 vs. 1–3).
Page 15.	Table S11.	Additional participant characteristics by k-means cluster.
Page 16.	Table S12.	Exploratory logistic regression using PCA-derived composite physical profile score (PC1) and discrimination comparison.

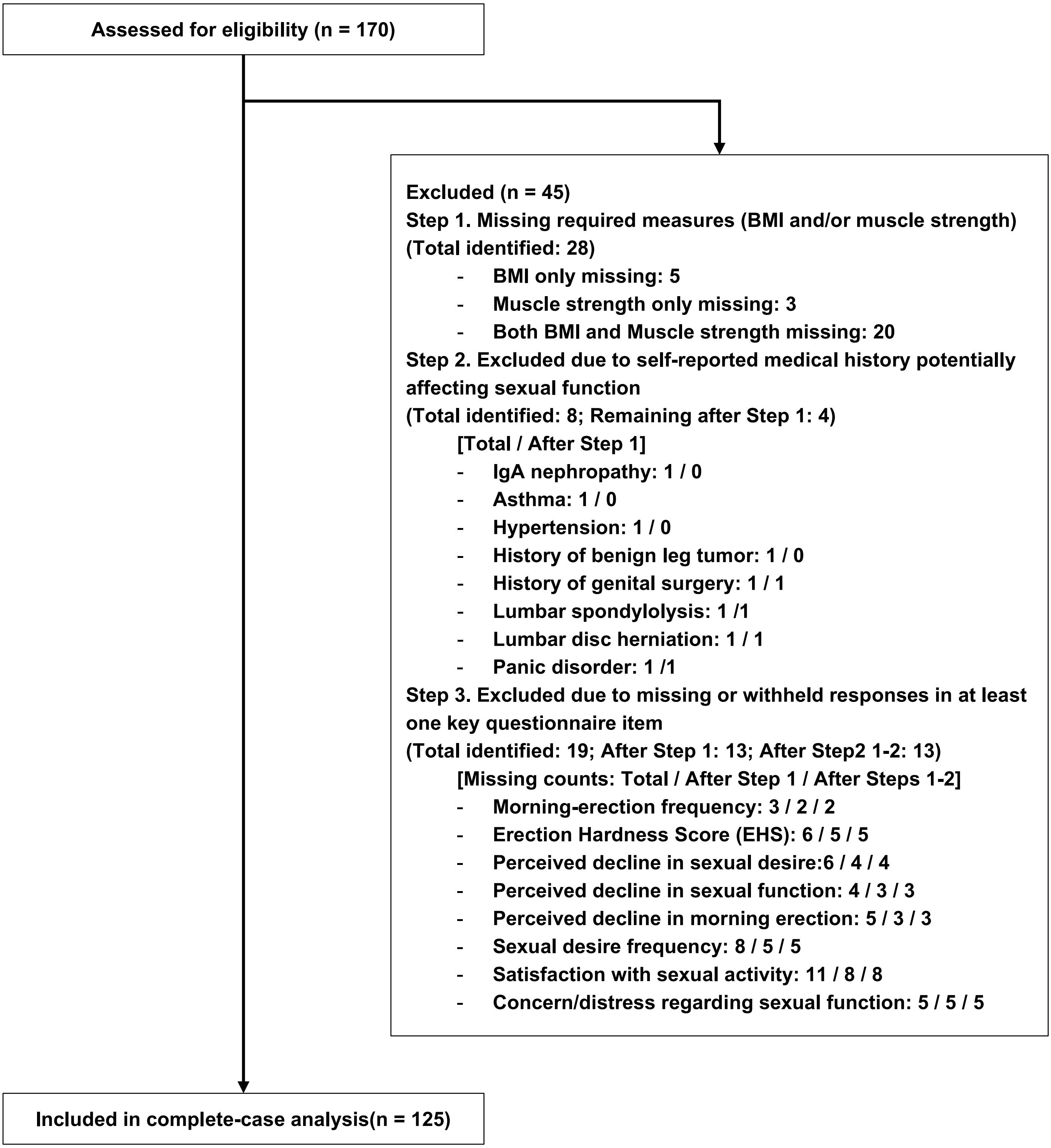

Figure S1. Flow diagram of participant enrollment. A total of 170 students were assessed for eligibility and 125 were included in the final analysis after exclusion.

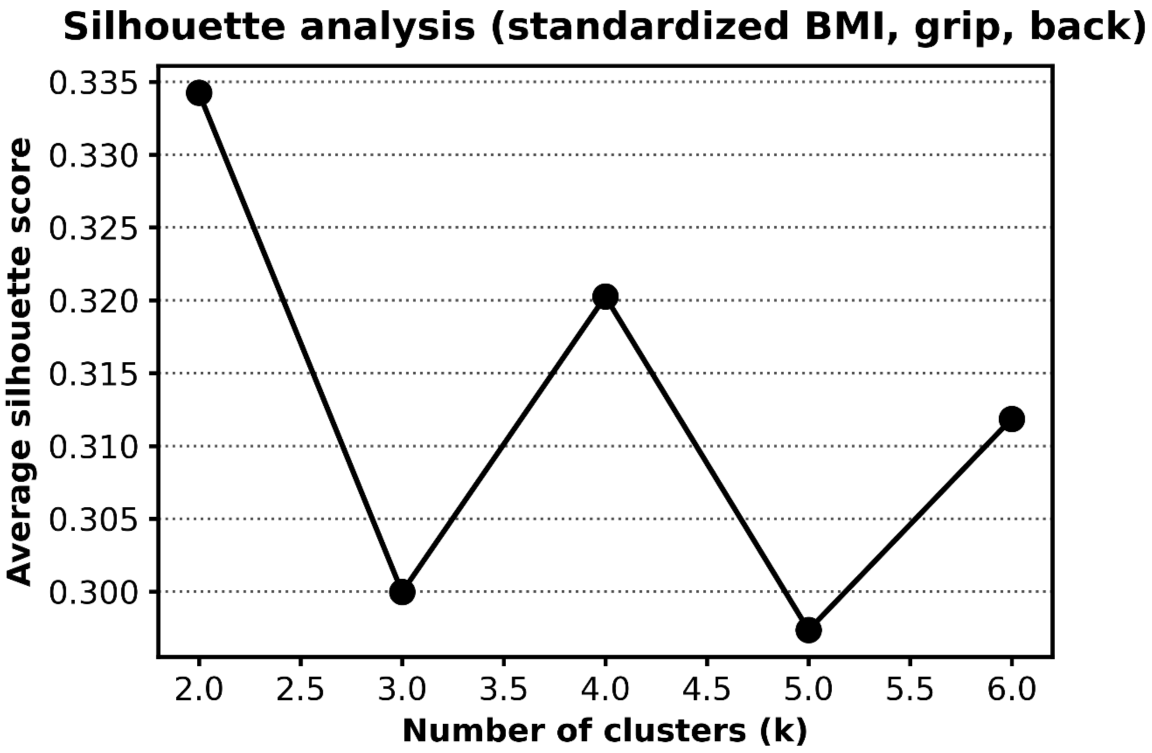

Figure S2. Silhouette analysis for k-means clustering. Average silhouette scores were calculated for k = 2–6 using standardized BMI, handgrip strength, and back muscle strength. The highest mean silhouette score supported the use of k = 2 for the exploratory clustering analysis.

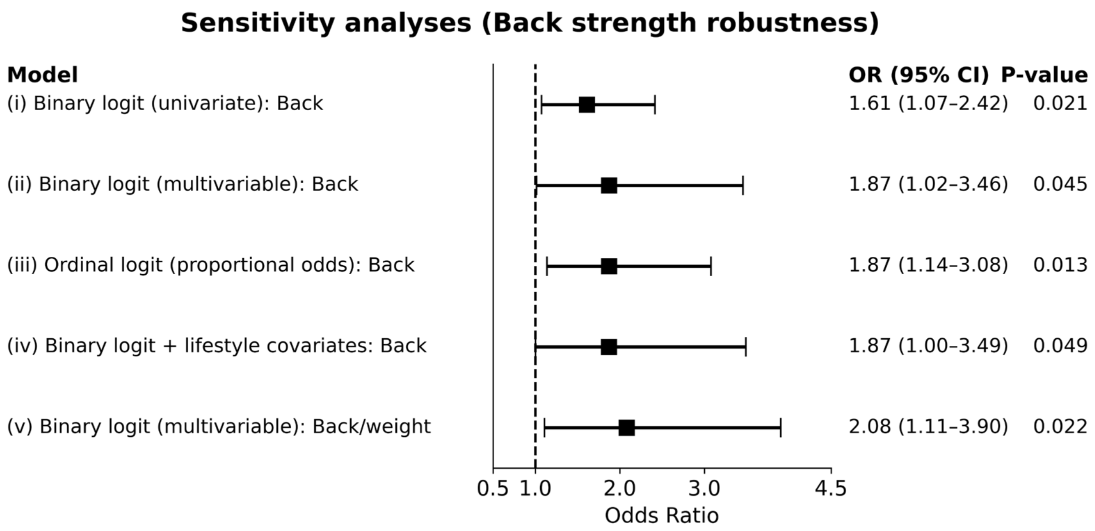

Figure S3. Sensitivity analyses for the association between back muscle strength and morning-erection frequency. Forest plot summarizing odds ratios (ORs) for back muscle strength across sensitivity analyses: (i) binary logistic regression (univariable), (ii) binary logistic regression (multivariable), (iii) ordinal logistic regression using the original six-point outcome (proportional odds model), (iv) multivariable logistic regression additionally adjusted for age, smoking status, alcohol consumption frequency, and total physical activity (per 1000 MET-min/week) derived from the IPAQ-SF, and (v) multivariable logistic regression using back muscle strength normalized to body mass. Rows (i) and (ii) reproduce the primary univariable and multivariable binary logistic models shown in Figure 1A–B and are presented here as reference estimates. Predictors were standardized (OR per 1 SD increase). Numeric OR (95% confidence interval) and p-values are displayed alongside the forest plot.

Table S1. Comparison of baseline characteristics between included and excluded participants.

Variable	Included (n = 125)	Excluded (n = 45)	p-value
Age (years)	20.09 ± 1.17	19.62 ± 0.72 (n = 45)	**0.013**
Height (cm)	171.11 ± 7.50	169.53 ± 5.56 (n = 20)	0.116
Body mass (kg)	65.49 ± 9.49	61.16 ± 6.08 (n = 21)	**0.011**
Body mass index (kg/m²)	22.33 ± 2.66	21.07 ± 1.68 (n = 20)	**0.037**
Handgrip strength (kg)	41.77 ± 7.61	43.37 ± 6.64 (n = 22)	0.273
Back muscle strength (kg)	113.12 ± 26.20	118.41 ± 17.40 (n = 22)	0.465
Morning-erection frequency (1-6)	3.59 ± 1.66	3.60 ± 1.59 (n = 42)	0.931
Morning-erection frequency, n(%)		(n = 42)	0.589
Low (≤3)	74 (59.2%)	27 (64.3%)	
High (≥4)	51 (40.8%)	15 (35.7%)	
Erection Hardness Score (0-4)	3.73 ± 0.68	3.77 ± 0.58 (n = 39)	0.896
Erection Hardness Score, n(%)		(n = 39)	0.681
Low (Score 0-2)	7 (5.6%)	1 (2.6%)	
High (Score 3-4)	118 (94.4%)	38 (97.4%)	
Perceived decline in sexual function (1-5)	1.20 ± 0.54	1.20 ± 0.51 (n = 41)	0.976
Perceived decline in morning erection (1-5)	1.30 ± 0.63	1.27 ± 0.75 (n = 40)	0.500
Perceived decline in sexual desire (1-5)	1.38 ± 0.67	1.33 ± 0.58 (n = 39)	0.808
Sexual desire frequency (1-8)	2.89 ± 1.91	2.27 ± 1.76 (n = 37)	0.060
Satisfaction with sexual activity (1-5)	3.62 ± 0.91	3.68 ± 0.94 (n = 34)	0.706
Concern/distress regarding sexual function (1-4)	1.36 ± 0.69	1.25 ± 0.71 (n = 40)	0.174
Alcohol consumption frequency (days/week)	0.48 ± 0.87	0.49 ± 0.92 (n = 45)	0.862
Smoking status, n (%)		(n = 45)	0.065
Never	101 (80.8%)	40 (88.9%)	
Past	4 (3.2%)	3 (6.7%)	
Current	20 (16.0%)	2 (4.4%)	
Vigorous physical activity (MET-min/week)	1109.3 ± 1469.5	1938.7 ± 2306.6 (n = 45)	**0.039**
Moderate physical activity (MET-min/week)	375.6 ± 643.9	375.6 ± 643.9 (n = 45)	0.428
Walking (MET-min/week)	823.5 ± 1078.3	1027.8 ± 1332.6 (n = 45)	0.977
Total physical activity (MET-min/week)	2308.5 ± 2168.4	3290.0 ± 3175.6 (n = 45)	0.162
Sitting time on weekdays (min/day)	382.3 ± 233.7	340.0 ± 188.5 (n = 45)	0.452
Physical activity level (IPAQ), n (%)		(n = 45)	0.391
Low	44 (35.2%)	15 (33.3%)	
Moderate	44 (35.2%)	12 (26.7%)	
High	37 (29.6%)	18 (40.0%)	
Frequency of consuming three meals (days/week)	3.73 ± 2.62	4.56 ± 2.19 (n = 45)	0.054
Frequency of consuming balanced meals (days/week)	3.90 ± 2.02	4.31 ± 2.00 (n = 45)	0.252
Frequency of eating or snacking after 21:00	3.19 ± 1.77	2.98 ± 1.57 (n = 45)	0.472
Meal timing regularity		(n = 45)	0.649
Regular	18 (14.4%)	9 (20.0%)	
Sometimes irregular	65 (52.0%)	23 (51.1%)	
Irregular	42 (33.6%)	13 (28.9%)	

Values are presented as mean ± standard deviation (SD) or n (%). Included participants were complete cases without self-reported medical conditions potentially affecting male sexual function. Excluded participants comprised those with medical conditions and/or missing/withheld responses (including "prefer not to answer") for questionnaire or measurement variables. For excluded participants, variable-specific (available-case) denominators are shown in parentheses because missingness was not uniform across variables. P-values were obtained using the Brunner-Munzel test for continuous variables. For categorical variables, Barnard's exact test was used for 2×2 contingency tables, and Fisher's exact test was used for 2×3 contingency tables.

Table S2. Comparison of sleep habits between included and excluded participants.

Variable	Included (n = 125)	Excluded (n = 45)	p-value
Bedtime on weekdays (HH:MM ± SD (minute))	00:40 ± 87 (n = 117)	00:37 ± 50 (n = 41)	0.323
Bedtime on weekdays, n (%)	(n = 117)	(n = 41)	0.588
≤ 00:29	42 (35.9%)	17 (41.5%)	
00:30–01:29	46 (39.3%)	17 (41.5%)	
≥ 01:30	29 (26.5%)	7 (17.1%)	
Bedtime on weekends (HH:MM ± SD (minute))	01:25 ± 92 (n = 117)	01:01 ± 90 (n = 41)	0.229
Bedtime on weekends, n (%)	(n = 117)	(n = 41)	0.683
≤ 00:29	31 (26.5%)	13 (31.7%)	
00:30–01:29	37 (31.6%)	14 (34.1%)	
≥ 01:30	49 (41.9%)	14 (34.1%)	
Wake-up time on weekdays (HH:MM ± SD (minute))	07:16 ± 52 (n = 117)	07:10 ± 44 (n = 41)	0.353
Wake-up time on weekdays, n (%)	(n = 117)	(n = 41)	0.596
≤ 06:59	24 (20.5%)	11 (26.8%)	
07:00–07:59	63 (53.8%)	22 (53.7%)	
≥ 08:00	30 (25.6%)	8 (19.5%)	
Wake-up time on weekends (HH:MM ± SD (minute))	09:27 ± 107 (n = 117)	09:10 ± 96 (n = 41)	0.294
Wake-up time on weekends, n (%)	(n = 117)	(n = 41)	0.600
≤ 06:59	4 (3.4%)	1 (2.4%)	
07:00–07:59	8 (6.8%)	5 (12.2%)	
≥ 08:00	105 (89.7%)	35 (85.4%)	
Sleep duration on weekdays (hours)	6.59 ± 1.38 (n = 117)	6.56 ± 1.00 (n = 41)	0.785
Sleep duration on weekends (hours)	8.19 ± 1.55 (n = 117)	8.15 ± 1.30(n = 41)	0.850
Social jetlag (mean (minutes) ± SD (minute))	83.8 ± 68.8 (n = 117)	72.4 ± 62.9 (n = 41)	0.396

Values are presented as mean ± standard deviation (SD) or n (%). P-values were obtained using the Brunner-Munzel test for continuous variables. For categorical variables, Fisher’s exact test was used. Self-reported and completed but invalid values (e.g., negative sleep duration, bedtime/wake-up times falling within university hours on weekdays) were treated as missing values.

Table S3. Additional participant characteristics and sexual function variables stratified by morning-erection frequency group (n = 125).

Variable	Total (n = 125)	Low (1–3) (n = 74)	High (4–6) (n = 51)	p-value
Height (cm)	171.11±7.50	171.34±8.05	170.77±6.68	0.417
Body mass (kg)	65.49±9.49	65.41±10.79	65.60±7.32	0.838
EHS	3.73 ± 0.68	3.64 ± 0.80	3.86 ± 0.40	0.080
EHS, n (%)				0.165
Low (Score 0-2)	7 (5.6%)	6 (8.1%)	1 (2.0%)	
High (Score 3-4)	118 (94.4%)	68 (91.9%)	50 (98.0%)	
Perceived decline in sexual function (1-5)	1.20 ± 0.54	1.24 ± 0.54	1.14 ± 0.53	0.080
Perceived decline in morning erection (1-5)	1.30 ± 0.63	1.35 ± 0.65	1.24 ± 0.59	0.256
Perceived decline in sexual desire (1-5)	1.38 ± 0.67	1.43 ± 0.74	1.29 ± 0.54	0.313
Sexual desire frequency (1-8)	2.89 ± 1.91	2.84 ± 1.97	2.96 ± 1.83	0.666
Satisfaction with sexual activity (1-5)	3.62 ± 0.91	3.54 ± 0.89	3.73 ± 0.92	0.324
Concern/distress regarding sexual function (1-4)	1.36 ± 0.69	1.32 ± 0.66	1.41 ± 0.73	0.522
Vigorous physical activity (MET-min/week)	1109.3 ± 1469.5	1218.4 ± 1597.5	951.1 ± 1259.5	0.469
Moderate physical activity (MET-min/week)	375.6 ± 643.9	406.8 ± 666.5	330.4 ± 613.3	0.358
Walking (MET-min/week)	823.5 ± 1078.3	660.2 ± 815.8	1060.5 ± 1347.4	0.368
Total physical activity (MET-min/week)	2308.5 ± 2168.4	2285.4 ± 2229.0	2342.0 ± 2098.7	0.769
Sitting time on weekdays (min/day)	382.3 ± 233.7	363.6 ± 238.2	409.4 ± 226.6	0.182
Physical activity level (IPAQ), n (%)				0.566
Low	44 (35.2%)	25 (33.8%)	19 (37.3%)	
Moderate	44 (35.2%)	29 (39.2%)	15 (29.4%)	
High	37 (29.6%)	20 (27.0%)	17 (33.3%)	
Frequency of consuming three meals (days/week)	3.73 ± 2.62	3.76 ± 2.64	3.69 ± 2.63	0.867
Frequency of consuming balanced meals (days/week)	3.90 ± 2.02	3.89 ± 2.01	3.90 ± 2.06	0.948
Frequency of eating or snacking after 21:00	3.19 ± 1.77	3.19 ± 1.83	3.20 ± 1.69	0.800
Meal timing regularity, n (%)				0.520
Regular	18 (14.4%)	13 (17.6%)	5 (9.8%)	
Sometimes irregular	65 (52.0%)	37 (50.0%)	28 (54.9%)	
Irregular	42 (33.6%)	24 (32.4%)	18 (35.3%)	

Values are presented as mean ± standard deviation (SD) or n (%). P-values were obtained using the Brunner-Munzel test for continuous variables. For categorical variables, Barnard's exact test was used for 2×2 contingency tables, and Fisher's exact test was used for 2×3 contingency tables. EHS, Erection Hardness Score.

Table S4. Sleep habits stratified by morning-erection frequency group among included participants (n = 125).

Variable	Total (n = 125)	Low (1–3) (n = 74)	High (4–6) (n = 51)	p-value
Bedtime on weekdays (HH:MM ± SD (minute))	00:40 ± 87	00:24 ± 96	01:03 ± 65	**0.027**
Bedtime on weekdays, n (%)				0.396
≤ 00:29	42 (35.9%)	20 (28.6%)	9 (19.1%)	
00:30–01:29	46 (39.3%)	28 (40.0%)	18 (38.3%)	
≥ 01:30	29 (26.5%)	22 (31.4%)	20 (42.6%)	
Bedtime on weekends (HH:MM ± SD (minute))	01:25 ± 92	01:07 ± 95	01:28 ± 87	0.646
Bedtime on weekends, n (%)				0.799
≤ 00:29	31 (26.5%)	13 (18.6%)	10 (21.3%)	
00:30–01:29	37 (31.6%)	21 (30.0%)	16 (34.0%)	
≥ 01:30	49 (41.9%)	36 (51.4%)	21 (44.7%)	
Wake-up time on weekdays (HH:MM ± SD (minute))	07:16 ± 52	07:12 ± 51	07:21 ± 54	0.300
Wake-up time on weekdays, n (%)				0.357
≤ 06:59	24 (20.5%)	14 (20.0%)	10 (21.3%)	
07:00–07:59	63 (53.8%)	41 (58.6%)	22 (46.8%)	
≥ 08:00	30 (25.6%)	15 (21.4%)	15 (31.9%)	
Wake-up time on weekends (HH:MM ± SD (minute))	09:27 ± 107	09:22 ± 86	09:34 ± 132	0.993
Wake-up time on weekends, n (%)				0.397
≤ 06:59	4 (3.4%)	2 (2.9%)	2 (4.3%)	
07:00–07:59	8 (6.8%)	3 (4.3%)	5 (10.6%)	
≥ 08:00	105 (89.7%)	65 (92.9%)	40 (85.1%)	
Sleep duration on weekdays (hours)	6.59 ± 1.38	6.79 ± 1.62	6.30 ± 0.88	0.146
Sleep duration on weekends (hours)	8.19 ± 1.55	8.26 ± 1.60	8.10 ± 1.50	0.706
Social jetlag (mean (minutes) ± SD (minute))	83.8 ± 68.8	86.4 ± 65.2	80.0 ± 74.4	0.362

Values are presented as mean ± standard deviation (SD) or n (%). P-values were obtained using the Brunner-Munzel test for continuous variables. Statistical tests were not performed for the categorical variables of bedtime and wake-up times, as these categories were arbitrarily defined for descriptive purposes and p-values are sensitive to threshold selection. Self-reported values judged to be invalid (e.g., negative sleep duration, or weekday bedtime/wake-up times falling within typical university hours) were treated as missing. Therefore, the available-case sample sizes for the sleep-related variables were n = 117 overall, n = 70 in the Low group, and n = 47 in the High group.

Table S5. Distribution of additional sexual function variables among participants (n = 125).

Variable / Score	N	%	
Perceived decline in sexual function			AMS item 5
1 (None)	107	85.6%	
2 (Mild)	12	9.6%	
3 (Moderate)	5	4.0%	
4 (Severe)	1	0.8%	
5 (Very severe)	0	0.0%	
Perceived decrease in morning erection			AMS item 16
1 (None)	97	77.6%	
2 (Mild)	19	15.2%	
3 (Moderate)	8	6.4%	
4 (Severe)	1	0.8%	
5 (Very Severe)	0	0.0%	
Perceived decline in sexual desire			AMS item 17
1 (None)	87	69.6%	
2 (Mild)	32	25.6%	
3 (Moderate)	4	3.2%	
4 (Severe)	1	0.8%	
5 (Very Severe)	1	0.8%	
Sexual desire frequency			Adapted from IIEF item 11 and EMAS-SFQ item 2
1 (Not at all)	47	37.6%	
2 (Once a month)	16	12.8%	
3 (2-3 times a month)	16	12.8%	
4 (Once a week)	14	11.2%	
5 (2-3 times a week)	20	16.0%	
6 (4-5 times a week)	8	6.4%	
7 (Once a day)	2	1.6%	
8 (Twice or more a day)	2	1.6%	
Satisfaction with sexual activity			Adapted from IIEF item 13 and EMAS-SFQ item 19
1 (Very Dissatisfied)	1	0.8%	
2 (Somewhat dissatisfied)	8	6.4%	
3 (Neither)	54	43.2%	
4 (Somewhat satisfied)	37	29.6%	
5 (Very Satisfied)	25	20.0%	
Concern/distress regarding sexual function			Adapted from EMAS-SFQ (summarizes item 8, 11, 17)
1 (Not at all)	94	75.2%	
2 (Hardly at all)	18	14.4%	
3 (A little)	12	9.6%	
4 (Very much)	1	0.8%	

Values are n (%). AMS: Aging Males' Symptoms questionnaire; IIEF: International Index of Erectile Function; EMAS-SFQ: European Male Ageing Study Sexual Function Questionnaire.

Table S6. Spearman's correlation coefficients among anthropometric strength and morning-erection frequency variables (n = 125).

Variable	Morning-erection frequency (rs, p)	EHS (rs, p)	BMI (rs, p)	Handgrip strength (rs, p)	Back muscle strength (rs, p)	Height (rs, p)	Body mass (rs, p)	Age (rs, p)
Morning-erection frequency	1.00 (−)	0.201 **(0.025)**	0.045 (0.621)	0.131 (0.147)	0.256 (**0.004**)	−0.114 (0.205)	−0.020 (0.827)	0.052 (0.564)
EHS		1.00 (−)	0.048 (0.593)	−0.082 (0.366)	−0.150 (0.096)	−0.049 (0.585)	0.007 (0.937)	0.048 (0.593)
BMI	–	–	1.00 (−)	0.056 (0.536)	0.184 (**0.040**)	−0.081 (0.367)	0.756 (**<0.001**)	0.018 (0.839)
Handgrip strength	–	–	–	1.00 (−)	0.668 (**<0.001**)	0.198 (**0.027**)	0.205 (**0.022**)	−0.074 (0.415)
Back muscle strength	–	–	–	–	1.00 (−)	0.221 (**0.013**)	0.315 (**<0.001**)	−0.087 (0.334)
Height	–	–	–	–	–	1.00 (−)	0.538 (**<0.001**)	0.171 (0.056)
Body mass	–	–	–	–	–	–	1.00 (−)	0.123 (0.172)
Age	–	–	–	–	–	–	–	1.00 (−)

EHS, Erection Hardness Score; BMI, body mass index.

Table S7. Spearman's correlation coefficients among sexual function variables (n = 125).

**Variable**	**Morning-erection frequency (rs, p)**	**EHS (rs, p)**	**Perceived decline in sexual function (rs, p)**	**Perceived decrease in morning erection (rs, p)**	**Perceived decline in sexual desire (rs, p)**	**Sexual desire frequency (rs, p)**	**Satisfaction with sexual activity (rs, p)**	**Concern regarding sexual function (rs, p)**
Morning-erection frequency	1.00 (−)	0.201 (**0.025**)	−0.142 (0.113)	−0.067 (0.458)	−0.077 (0.395)	0.015 (0.866)	0.063 (0.485)	0.060 (0.506)
EHS	–	1.00 (−)	−0.068 (0.454)	−0.031 (0.735)	−0.020 (0.825)	0.237 (**0.008**)	0.202 (**0.024**)	0.018 (0.844)
Perceived decline in sexual function	–	–	1.00 (−)	0.492 (**<.001**)	0.413 (**<.001**)	0.019 (0.831)	0.042 (0.639)	0.126 (0.160)
Perceived decrease in morning erection	–	–	–	1.00 (−)	0.365 (**<.001**)	−0.011 (0.900)	−0.081 (0.368)	0.154 (0.087)
Perceived decline in sexual desire	–	–	–	–	1.00 (−)	−0.078 (0.386)	−0.179 (**0.046**)	0.189 (0.035)
Sexual desire frequency	–	–	–	–	–	1.00 (−)	0.240 (**0.007**)	0.214 (**0.016**)
Satisfaction with sexual activity	–	–	–	–	–	–	1.00 (−)	−0.098 (0.278)
Concern regarding sexual function	–	–	–	–	–	–	–	1.00 (−)

EHS, Erection Hardness Score.

Table S8. Spearman's correlation coefficients among anthropometric strength and additional sexual variables (n = 125).

Variable	BMI	Hand-grip strength	Back muscle strength	Height	Body mass	Age
Perceived decline in sexual function	−0.066 (0.462)	−0.037 (0.679)	−0.036 (0.690)	0.113 (0.208)	−0.001 (0.991)	0.012 (0.897)
Perceived decrease in morning erection	−0.009 (0.925)	0.065 (0.472)	0.039 (0.665)	−0.029 (0.751)	−0.002 (0.985)	−0.009 (0.918)
Perceived decline in sexual desire	−0.128 (0.156)	0.114 (0.207)	0.008 (0.927)	0.025 (0.778)	−0.105 (0.245)	0.043 (0.636)
Frequency of sexual desire	0.141 (0.118)	0.099 (0.271)	−0.020 (0.821)	−0.076 (0.401)	0.100 (0.268)	0.048 (0.597)
Satisfaction with sexual activity	0.024 (0.788)	−0.080 (0.377)	−0.036 (0.687)	0.170 (0.058)	0.113 (0.211)	0.026 (0.775)
Concern regarding sexual function	0.072 (0.425)	−0.000 (0.997)	0.039 (0.665)	−0.062 (0.494)	0.023 (0.800)	0.060 (0.506)

BMI, body mass index.

Table S9. Sensitivity analyses across alternative dichotomizations of morning-erection frequency.

Outcome definition (High vs Low)	Model	OR (95% CI) per 1 SD	p-value	AUC (95% CI)
Score 6 (n=25)	Back-only model	1.41 (0.86–2.31)	0.177	0.570 (0.447–0.694)
vs 1–5 (n=100)	BMI+grip+back model	1.28 (0.62–2.61)	0.504	0.575 (0.453–0.698)
Scores 5–6 (n=46)	Back-only model	1.46 (0.98–2.18)	0.066	0.595 (0.493–0.697)
vs 1–4 (n=79)	BMI+grip+back model	1.54 (0.84–2.80)	0.162	0.597 (0.495–0.699)
Scores 4–6 (n=51)	Back-only model	1.61 (1.07–2.42)	**0.021**	0.625 (0.525–0.724)
vs 1–3 (n=74)	BMI+grip+back model	1.88 (1.02–3.47)	**0.045**	0.633 (0.534–0.732)
Scores 3–6 (n=89)	Back-only model	1.76 (1.18–2.63)	**0.006**	0.673 (0.572–0.775)
vs 1–2 (n=36)	BMI+grip+back model	2.23 (1.15–4.32)	**0.017**	0.682 (0.583–0.781)
Scores 2–6 (n=113)	Back-only model	1.83 (1.08–3.11)	**0.026**	0.694 (0.543–0.844)
vs 1 (n=12)	BMI+grip+back model	2.61 (1.11–6.17)	**0.028**	0.686 (0.533–0.839)

For the primary dichotomization (scores 4–6 vs 1–3), the Back-only and BMI+grip+back rows reproduce the models shown in Figure 1A (Back-only, OR 1.61) and Figure 1B (BMI+grip+back, OR 1.88), respectively. AUC, area under the receiver operating characteristic curve; OR, odds ratio; CI, confidence interval.

Table S10. Univariable associations of age and lifestyle variables with high morning erection frequency (scores 4–6 vs. 1–3).

Variable	OR (95% CI)	p-value
Age (per 1 year)	1.04 (0.76–1.41)	0.814
Alcohol consumption frequency (per 1 day/week)	1.12 (0.74–1.68)	0.597
Ever smoker (vs never)	1.05 (0.42–2.58)	0.923
Vigorous physical activity (per 1000 MET-min/week)	0.90 (0.71–1.15)	0.410
Moderate physical activity (per 1000 MET-min/week)	0.80 (0.49–1.30)	0.373
Walking (per 1000 MET-min/week)	1.25 (1.01–1.55)	**0.037**
Total physical activity (per 1000 MET-min/week)	1.05 (0.93–1.19)	0.400
Sitting time on weekdays (per 60 min/day)	1.05 (0.96–1.15)	0.282
IPAQ physical activity level: Moderate (vs Low)	0.68 (0.29–1.61)	0.282
IPAQ physical activity level: High (vs Low)	1.12 (0.46–2.70)	0.803

Odds ratios (ORs) are shown with 95% confidence intervals (CIs).

Table S11. Additional participant characteristics by k-means cluster.

Variable	Cluster 1 (n = 41)	Cluster 2 (n = 84)	p-value
Age (years)	20.17 ± 1.46	20.05 ± 1.00	0.935
Height (cm)	169.31 ± 8.76	171.98 ± 6.68	0.254
Body mass (kg)	59.06 ± 8.12	68.62 ± 8.52	**<0.001**
Erection Hardness Score (0-4)	3.83 ± 0.44	3.68 ± 0.76	0.377
Erection Hardness Score, n (%)			0.425
Low (Score 0-2)	1 (2.4%)	6 (7.1%)	
High (Score 3-4)	40 (97.6%)	78 (92.9%)	
Perceived decline in sexual function (1–5)	1.34 ± 0.76	1.13 ± 0.37	0.236
Perceived decline in morning erection (1–5)	1.24 ± 0.58	1.33 ± 0.65	0.342
Perceived decline in sexual desire (1–5)	1.39 ± 0.80	1.37 ± 0.60	0.665
Sexual desire frequency (1–8)	2.71 ± 1.86	2.98 ± 1.93	0.425
Satisfaction with sexual activity (1–5)	3.71 ± 0.81	3.57 ± 0.95	0.557
Concern/distress regarding sexual function (1–4)	1.27 ± 0.71	1.40 ± 0.68	0.089
Alcohol consumption frequency (days/week)	0.39 ± 0.70	0.52 ± 0.94	0.534
Smoking status, n (%)			0.168
Never	33 (80.5%)	68 (81.0%)	
Past	3 (7.3%)	1 (1.2%)	
Current	5 (12.2%)	15 (17.9%)	
Vigorous physical activity (MET-min/week)	1121.95 ± 1329.48	1126.00 ± 1622.85	0.485
Moderate physical activity (MET-min/week)	668.29 ± 1304.95	298.48 ± 595.67	**0.015**
Walking (MET-min/week)	1114.76 ± 1823.07	1117.48 ± 2099.60	0.093
Total physical activity (MET-min/week)	2905.00 ± 3230.99	2541.96 ± 2769.61	0.304
Sitting time on weekdays (min/day)	332.20 ± 213.44	406.79 ± 240.42	0.071
Physical activity level (IPAQ), n (%)			0.286
Low	11 (26.8%)	33 (33.9%)	
Moderate	18 (43.9%)	26 (31.0%)	
High	12 (29.3%)	25 (29.8%)	

Values are presented as mean ± standard deviation or n (%). P values were calculated using the Brunner–Munzel test for continuous variables. For categorical variables, Barnard's exact test was used for 2×2 contingency tables, and Fisher's exact test was used for 2×3 contingency tables. Clusters were defined using standardized body mass index, handgrip strength, and back muscle strength (k = 2); variables in this table were not used to define clusters and are provided descriptively.

Table S12. Exploratory logistic regression using PCA-derived composite physical profile score (PC1) and discrimination comparison.

Model	OR (95% CI) per 1 SD	p-value	AUC (95% CI)
Back-only model (univariable)	1.61 (1.07–2.42)	**0.021**	0.625 (0.525–0.724)
BMI+grip+back model	1.88 (1.02–3.47)	**0.045**	0.633 (0.534–0.732)
PC1 composite score	1.48 (1.00–2.20)	**0.049**	0.611 (0.512–0.711)

The outcome was the primary dichotomization (scores 4–6 vs 1–3). Predictors were z-score standardized; ORs are per 1 SD increase. Back-only and BMI+grip+back rows report the OR for back muscle strength and reproduce the models shown in Figure 1A (Back-only OR 1.61) and Figure 1B (BMI+grip+back OR 1.88), respectively; these rows are provided as benchmarks for AUC comparisons. PC1 is the first principal component derived from standardized BMI, handgrip strength, and back muscle strength (59.0% variance explained; oriented so that higher values indicate higher back muscle strength). AUC 95% CIs were computed using DeLong's method. AUC, area under the receiver operating characteristic curve; OR, odds ratio; CI, confidence interval; BMI, body mass index; SD, standard deviation.
